# Long-Term Outcomes of Stent Relining for the Management of High-Pressure Endoleaks With Progressive Sac Expansion After Endovascular Aneurysm Repair (EVAR)

**DOI:** 10.7759/cureus.94685

**Published:** 2025-10-15

**Authors:** Mahmoud Bakheet, Yasser Elsayed, Ayesha Joorawan, Shahana Farook, Bilal Rawshdeh, Mohamed Banihani

**Affiliations:** 1 Vascular Surgery, Royal Preston Hospital, Preston, GBR

**Keywords:** aneurysm sac expansion, endoleak management, endovascular aortic repair, relining stent grafts, sac stabilization

## Abstract

Background

High-pressure endoleaks (Types I, III, and V) with progressive sac expansion remain a significant challenge following endovascular aortic repair (EVAR). Stent graft relining, with or without adjunctive procedures such as femoral crossover bypass, embolization, or chimney stents, has emerged as an important intervention. However, data on long-term outcomes remain limited. We evaluated the efficacy and durability of relining in this patient cohort.

Methods

We performed a retrospective analysis of all patients treated with EVAR stent relining for Type I, III, or V endoleaks with progressive sac growth (>5mm) at a regional vascular center between 2014 and 2024. Data included demographics, comorbidities, aneurysm characteristics, type of endoleak, mode of presentation, technical success, sac stabilization, reintervention rates, and survival outcomes.

Results

Seventy-nine patients had significant endoleaks, of whom 32 (40.5%) underwent relining (13 Type I, nine Type III, 10 Type V). Mean time to endoleak was 57 months. Technical success was 100%. Sac stabilization was achieved in 87.5% of patients at a mean follow-up of 48 months. The reintervention rate was 15.6%. Twenty-five patients (78%) were treated electively, while seven (22%) underwent emergency relining for rupture. There was no 30-day mortality in either group. At one year, survival was 100% in the elective group versus 71% in the emergency group (p=0.01), falling to 57% at two years (p=0.002). Emergency relining was associated with a 5.6-fold higher risk of sac stabilization failure compared with elective repair.

Conclusion

Stent relining is a highly effective treatment for high-pressure endoleaks with sac expansion, achieving excellent technical success and durable sac stabilization when performed electively. However, outcomes are significantly worse in emergency settings, with higher failure and mortality rates. These findings strongly support prioritizing elective relining for progressive endoleaks to optimize long-term outcomes.

## Introduction

Endovascular aortic repair (EVAR) has transformed the management of abdominal aortic aneurysms (AAAs) since its introduction in the 1990s, offering reduced perioperative mortality compared to open repair [1]. However, this minimally invasive approach introduces unique complications, most notably endoleaks with persistent blood flow outside the stent graft but within the aneurysm sac, which occur in 20-30% of cases and remain the Achilles’ heel of EVAR [2].

Endoleaks are classified into five types, with Type I (inadequate seal at graft ends), III (component separation or fabric tear), and V (“endotension”) being particularly concerning due to their association with continued sac expansion and rupture risk [3]. Unlike Type II endoleaks (branch vessel backflow), which often resolve spontaneously, these high-pressure endoleaks typically require intervention when accompanied by sac growth >5 mm [4]. Progressive expansion occurs in approximately 60% of untreated cases, carrying a 10-20% annual rupture risk once the sac exceeds 6 cm [5].

Management options include endovascular reintervention (relining, extensions, embolization), open conversion, or conservative monitoring. Stent graft relining, in which a new endograft is deployed within the existing one, has emerged as a preferred strategy for suitable anatomy, combining the minimally invasive advantages of EVAR with the ability to seal leaks and reinforce weakened components [6]. This approach theoretically reduces sac pressure while maintaining perfusion to critical branches, but long-term durability data remain scarce beyond five years of follow-up [7].

Current literature presents conflicting evidence. Some studies report 80-90% technical success with relining but note 15-25% reintervention rates [8], while others demonstrate superior outcomes compared to open conversion, particularly in elderly comorbid patients [9]. The Vascular Surgery Society’s 2022 guidelines conditionally recommend relining for Type I and III endoleaks (Class IIa evidence) but emphasize the need for more robust outcome data [10]. Notably, most available studies combine all endoleak types or lack stratification by urgency (elective vs. emergency), masking potential outcome disparities [11].

In this study, we analyzed a decade of experience with stent relining specifically for Type I, III, and V endoleaks with documented sac expansion. We focused on three key questions: (i) What are the technical success and sac stabilization rates? (ii) How do outcomes differ between elective and emergency presentations? and (iii) What factors predict reintervention or mortality? Our findings aim to refine patient selection and timing for this increasingly utilized procedure.

## Materials and methods

Study design and setting

We conducted a retrospective, single-center cohort study at a high-volume regional vascular referral center, Royal Preston Hospital, Lancashire Teaching Hospitals NHS Foundation Trust, Preston, United Kingdom. Consecutive patients treated between January 2014 and December 2024 were identified from a prospectively maintained vascular surgery database and verified against electronic medical records (EMR), operative notes, multidisciplinary team (MDT) records, and imaging archives. Ethical approval was not required because this study used fully anonymized retrospective data collected as part of routine clinical care and involved no patient contact or intervention, in line with institutional policy. The study adhered to the principles of the Declaration of Helsinki. The requirement for individual informed consent was waived due to the retrospective design and use of anonymized data.

Patient selection and eligibility

All patients who underwent EVAR at our center and were subsequently treated with stent-graft relining for a high-pressure endoleak were screened for eligibility. Patients were eligible if computed tomography angiography (CTA) demonstrated a Type I, Type III, or Type V (endotension) endoleak in association with progressive sac expansion, defined a priori as an increase of ≥5 mm in the maximum anteroposterior or transverse sac diameter on serial surveillance imaging. For Type V specifically, endotension was defined as sac growth in the absence of any detectable Type I-IV endoleak, in accordance with the Society for Vascular Surgery (SVS) practice guidelines [3]. Treatment with stent-graft relining, with or without adjunctive endovascular procedures, was required for inclusion. We excluded patients with isolated Type II endoleaks managed conservatively or by non-relining interventions, those who underwent open surgical conversion at the index reintervention, those with incomplete clinical or imaging follow-up of less than 12 months unless death occurred earlier, and cases in which sac expansion was attributable to proven graft infection.

Data collection and definitions

Data were extracted independently by two reviewers, and discrepancies were resolved by consensus. Baseline variables included age, sex, smoking status, hypertension, diabetes, chronic obstructive pulmonary disease, ischemic heart disease, antiplatelet/anticoagulant use, and initial abdominal aortic aneurysm (AAA) diameter (pre-EVAR). Endoleak variables included type (I/III/V), time from EVAR to endoleak detection, maximum sac diameter immediately pre-relining, and sac expansion rate (mm/year). Procedural variables included device/relining strategy, adjuncts (e.g., embolization, iliac limb extension, fem-fem crossover, chimney/periscope stents), and urgency (elective vs emergency/rupture).

Technical success was defined as successful deployment of the intended device with angiographic resolution of the causative endoleak or correction of the presumed mechanism of endotension, without intra-procedural mortality. Sac stabilization was defined a priori as ≤5 mm change in maximum sac diameter on subsequent annual imaging after the index relining. Failure of stabilization was defined as >5 mm growth at any time point after relining. Reinterventions and mortality (30-day, one-year, and overall) were recorded.

Imaging and follow-up

Surveillance followed local protocols aligned with contemporary ESVS guidance [2]: CTA at one month and 12 months post-relining, then annually thereafter; duplex ultrasound was used for patients with significant renal impairment. All CTA measurements and endoleak classifications were performed on a clinically licensed PACS (Picture Archiving and Communication System)/workstation (Sectra PACS; Sectra AB, Linköping, Sweden). When present, discrepancies were resolved by consensus or senior radiology review.

Statistical analysis

Analyses were performed using IBM SPSS Statistics for Windows, Version 28.0 (IBM Corp., Armonk, New York, United States). Normality of continuous variables was assessed with the Shapiro-Wilk test. Continuous data are reported as mean ± standard deviation (SD) or median (interquartile range (IQR)) as appropriate and were compared using the independent-samples t test or Mann-Whitney U test. Categorical variables are presented as counts (percentages) and were compared using the χ² test or Fisher’s exact test when expected cell counts were <5. For survival proportions reported at fixed time points (30-day, one-year, two-year), group differences were tested with χ² or Fisher’s exact test as appropriate. Two-sided p < 0.05 was considered statistically significant.

## Results

Patient cohort overview

Of 79 patients with significant endoleaks, 32 underwent relining (40.5%) and formed the study group (all male, mean age 77 ± 8 years). Baseline characteristics are shown in Table 1.

**Table 1:** Baseline characteristics *significant difference (p < 0.05) between elective and emergency groups. AAA: abdominal aortic aneurysm

Variable	Overall (n=32)	Elective (n=25)	Emergency (n=7)
Age (years), mean±SD	77 ± 8	76 ± 7	82 ± 6
Smoking history, n (%)	18 (56%)	14 (56%)	4 (57%)
Coronary artery disease, n (%)	11 (34%)	7 (28%)	4 (57%)*
Diabetes, n (%)	7 (22%)	5 (20%)	2 (29%)
Initial AAA size (mm), mean±SD	75 ± 12	73 ± 11	82 ± 14*
Time to endoleak (mo), mean±SD	57 ± 32	62 ± 30	39 ± 28*

Endoleak characteristics and relining outcomes

**Figure 1:**
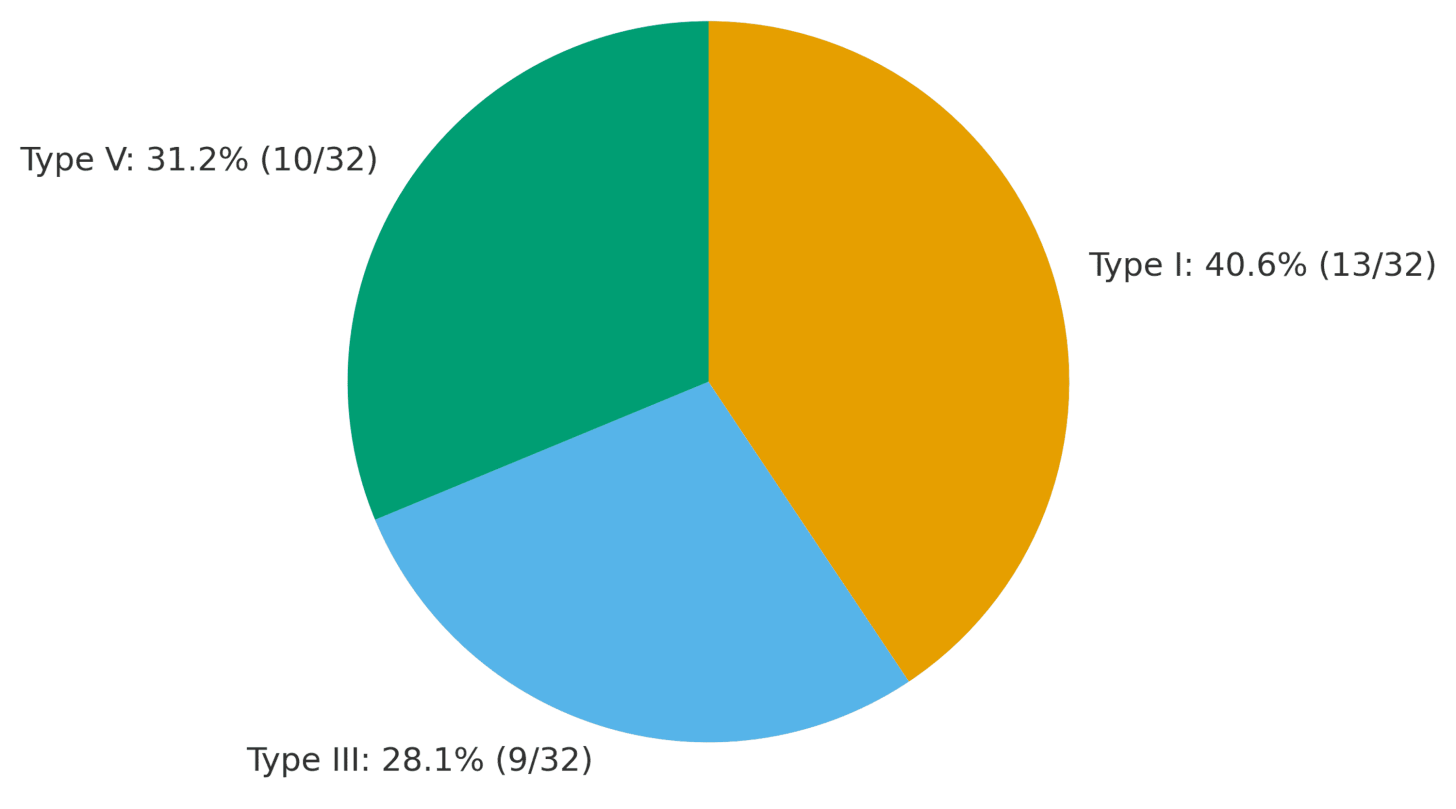
Endoleak type distribution Numbers indicate patient counts (N) with percentages of the total cohort (N = 32): Type I 41% (13/32), Type III 28% (9/32), Type V 31% (10/32). Endoleak classification according to the Society for Vascular Surgery practice guidelines [3].

Technical success was achieved in 100% of cases, with all patients demonstrating intraoperative endoleak resolution. Sac stabilization was observed in 28/32 patients (87.5%) at a mean follow-up of 48 months. Five patients (15.6%) required reinterventions (three for new endoleaks and two for graft limb occlusion). Outcomes by endoleak type are summarized in Table 2. Endoleak type distribution is demonstrated in Figure 1.

**Table 2:** Outcomes by endoleak type

Outcome	Type I (n=13), n (%)	Type III (n=9), n (%)	Type V (n=10), n (%)
Sac stabilization	11 (85%)	8 (89%)	9 (90%)
Reintervention	3 (23%)	1 (11%)	1 (10%)

Survival outcomes

All patients survived the first month after surgery, whether treated electively or in the emergency setting. However, survival at one year was significantly lower in the emergency group (71%) compared with the elective group (100%, p=0.01). At two years, survival dropped further to 57% in the emergency group, while remaining 100% in the elective group (p=0.002). Survival rates by urgency of repair are detailed in Table 3.

**Table 3:** Survival rates by repair urgency Note: Fisher’s exact test was used for categorical comparisons due to small sample sizes. Test statistic values are not reported for Fisher’s exact test; instead, exact p-values are provided.

Time Point	Elective (n=25), n (%)	Emergency (n=7), n (%)	Statistical Test	Test Statistic	p-value
30-day	25/25 (100%)	7/7 (100%)	Fisher’s exact	–	1.00
1-year	25/25 (100%)	5/7 (71%)	Fisher’s exact	–	0.01
2-year	25/25 (100%)	4/7 (57%)	Fisher’s exact	–	0.002

Predictors of sac stabilization failure

**Figure 2:**
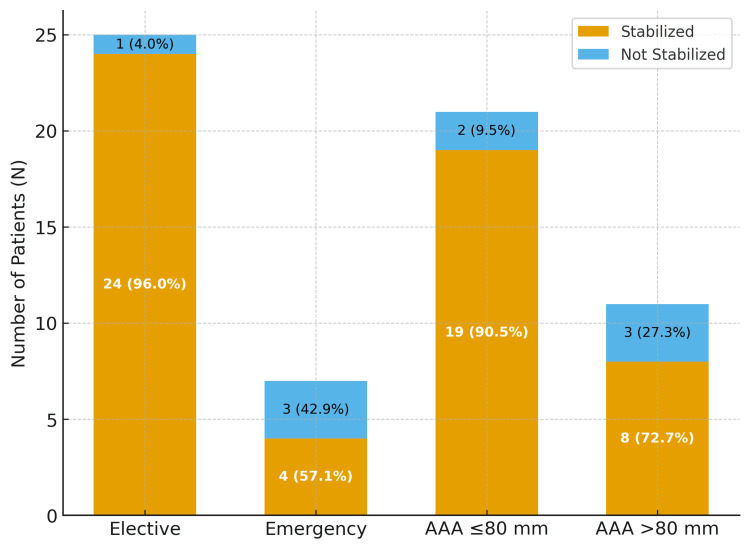
Predictors of sac stabilization failure Sac stabilization by group. Bars show “Stabilized” and “Not stabilized” with counts and percentages. “Stabilized” was defined a priori as ≤5 mm change in maximum sac diameter on subsequent annual imaging (see Methods). Subgroup totals: Elective n = 25, Emergency n = 7, AAA ≤ 80 mm n = 21, AAA > 80 mm n = 11 (total N = 32). AAA: abdominal aortic aneurysm

Emergency procedures demonstrated significantly worse outcomes, with 5.6 times higher odds of ongoing sac growth compared to elective cases (95%CI 1.3-24.1, p=0.02). Larger aneurysms (>80 mm) showed a trend toward failure (OR 2.1, p=0.12), though this did not reach statistical significance. No other clinical factors were associated with failure. Predictors of sac stabilization failure are illustrated in Figure 2.

## Discussion

Our unit’s experience with stent relining for endoleaks with sac expansion demonstrated three principal findings: (i) excellent technical success and sac stabilization rates, (ii) significant disparities between elective and emergency cases, and (iii) emergency presentation as the sole independent predictor of failure. These results refine current evidence in several key aspects.

Comparative efficacy of relining

Our 100% technical success rate aligns with prior studies of Type I and III endoleaks [6] and with the outcomes reported in recent relining series for complex endoleaks [12]. The 87.5% sac stabilization rate at 48 months parallels the 85% three-year success reported in contemporary series [13], suggesting improved durability with modern stent-grafts. Our reintervention rate (15.6%) was also lower than other case series [8], which may reflect stricter patient selection.

The superior outcomes for Type III versus Type I endoleaks (89% vs 85% stabilization) corroborate biomechanical studies showing greater seal zone challenges in proximal compared to component-separation failures [14]. This supports the ESVS guidelines’ emphasis on proximal seal optimization during initial EVAR [2].

The emergency-elective disparity

The 43% absolute survival difference at two years between elective (100%) and emergency (57%) cases mirrors IMPROVE trial data, which demonstrated a 32% higher mortality rate for ruptured EVAR [15]. Several factors likely contributed, including hemodynamic instability in ruptured AAA impairing stent-graft apposition [16], acute inflammation promoting endotension [17], limited endovascular options in rupture scenarios [9], and more liberal use of EVAR “outside IFU (Instructions for Use)” in life-saving emergencies. Notably, none of the patients treated with relining were fit for open repair even after recovery from rupture. Our finding that emergency relining carries 5.6-fold higher odds of failure (p=0.02) strongly supports the VSS guidelines’ Class IIa recommendation for early intervention [10].

Predictors of failure

Although aneurysm size greater than 80 mm showed a twofold increase in failure risk (p=0.12), this did not reach statistical significance. However, it aligns with finite-element analyses that demonstrate reduced stent adhesion in large thrombotic sacs [18]. Interestingly, comorbidities did not predict failure, suggesting that relining remains viable even in high-risk patients if performed electively.

Technical considerations

The 96% success in elective relining parallels outcomes reported from the latest Endurant II graft trials [19]. In emergency cases, proximal fixation techniques have been shown to enhance sealing and procedural stability in contemporary EVAR practice [20].

Limitations

This study has several limitations. It was conducted at a single center, which may limit generalizability to other practice settings. The retrospective nature of the study means that unmeasured confounders, such as subtle anatomic variations, could have influenced outcomes. The mean follow-up duration of 48 months provides valuable mid-term data but does not fully address very late failures beyond five years, which have been reported in other series. In addition, the exclusion of Type II endoleaks and open conversions may have led to an overestimation of success rates compared to all-comer EVAR populations. Future multicenter studies with extended follow-up are needed to validate these findings and further refine patient selection for stent relining.

## Conclusions

Stent relining is a safe and effective treatment for high-pressure endoleaks with progressive sac expansion after EVAR, achieving high technical success and sac stabilization rates at mid-term follow-up. Outcomes were markedly superior when the procedure was performed electively, with emergency cases showing substantially higher risks of sac growth and mortality.

These findings highlight the importance of strict post-EVAR surveillance and timely elective intervention for Type I, III, and V endoleaks. Future multicenter studies with larger cohorts and longer follow-up are warranted to confirm durability and refine patient selection criteria.
